# Health Professionals’ Attitudes Towards Using a Web 2.0 Portal for Child and Adolescent Diabetes Care: Qualitative Study

**DOI:** 10.2196/jmir.1152

**Published:** 2009-04-06

**Authors:** Cecilia Nordqvist, Lena Hanberger, Toomas Timpka, Sam Nordfeldt

**Affiliations:** ^5^Center for Medical Technology AssessmentDepartment of Medicine and Health SciencesLinköping UniversitySweden; ^4^Division of Child and Adolescent PsychiatryDepartment of Clinical and Experimental MedicineLinköping UniversitySweden; ^3^Department of Computer ScienceLinköping UniversitySweden; ^2^Division of PediatricsDepartment of Clinical and Experimental MedicineLinköping UniversitySweden; ^1^Division of Community MedicineDepartment of Medical and Health SciencesLinköping UniversitySweden

**Keywords:** Web 2.0, childhood chronic disease, health professionals, attitudes, type 1 diabetes

## Abstract

**Background:**

The Internet, created and maintained in part by third-party apomediation, has become a dynamic resource for living with a chronic disease. Modern management of type 1 diabetes requires continuous support and problem-based learning, but few pediatric clinics offer Web 2.0 resources to patients as part of routine diabetes care.

**Objectives:**

To explore pediatric practitioners’ attitudes towards the introduction of a local Web portal for providing young type 1 diabetes patients with interactive pedagogic devices, social networking tools, and locally produced self-care and treatment information. Opportunities and barriers related to the introduction of such systems into clinical practice were sought.

**Methods:**

Twenty clinicians (seven doctors, nine nurses, two dieticians, and two social welfare officers) from two pediatric diabetes teams participated in the user-centered design of a local Web 2.0 portal. After completion of the design, individual semi-structured interviews were performed and data were analyzed using phenomenological methods.

**Results:**

The practitioners reported a range of positive attitudes towards the introduction of a local Web 2.0 portal to their clinical practice. Most interviewees were satisfied with how the portal turned out, and a sense of community emerged during the design process and development of the portal’s contents. A complementary role was suggested for the portal within the context of health practice culture, where patients and their parents would be able to learn about the disease before, between, and after scheduled contacts with their health care team. Although some professionals expected that email communication with patients and online patient information would save time during routine care, others emphasized the importance of also maintaining face-to-face communication. Online peer-to-peer communication was regarded as a valuable function; however, most clinicians did not expect that the portal would be used extensively for social networking amongst their patients. There were no major differences in attitudes between different professions or clinics, but some differences appeared in relation to work tasks.

**Conclusions:**

Experienced clinical practitioners working in diabetes teams exhibited positive attitudes towards a Web 2.0 portal tailored for young patients with type 1 diabetes and their parents. The portal included provision of third-party information, as well as practical and social means of support. The practitioners’ early and active participation provides a possible explanation for these positive attitudes. The findings encourage close collaboration with all user groups when implementing Web 2.0 systems for the care of young patients with chronic diseases, particularly type 1 diabetes. The study also highlights the need for efforts to educate clinical practitioners in the use of Web publishing, social networking, and other Web 2.0 resources. Investigations of attitudes towards implementing similar systems in the care of adults with chronic diseases are warranted.

## Introduction

For individuals with a chronic health problem, the Internet has evolved from being a source for medical information retrieval (Web 1.0) to being a dynamic resource for living with a chronic disease, one that is created and maintained in part by third-party apomediation (Web 2.0) [1-3]. The broadened scope of information in the Web 2.0 context has been followed by a parallel evolution of information practices (eg, the introduction of new types of quality criteria for evaluating the presentation and trustworthiness of medical advice) [4]. In many respects, the concurrent development of medical information on the Internet towards both openness and control reflects the present transformation of health services organizations, where quality surveillance has become more and more important, generating increased participation, collaboration, and inter-organizational networking [5,6].

The Web 2.0 and open health service organization perspectives are equally applicable to the modern management of type 1 diabetes, since both possess a common denominator of focus on continuous support and problem-based learning [7,8]. For many patients, adolescence is a period during which diabetes care constitutes a more or less daily struggle with undesirable blood glucose levels and the risk of complications [9,10]. Long-term evaluations of diabetes treatment programs emphasize the importance of metabolic control [7,11,12]. Finding the means to educate and support young patients and their families is therefore of the utmost importance. Recent research focusing on patient views suggests that pediatric diabetes care needs improvement regarding patient information and access to care [13]. Previous studies indicate that successful use of interactive telecare and Internet-based methods may increase access to health services, enhance patient education, and improve the quality of diabetes care [14,15]. Internet-based interventions have been reported to influence diabetic patients’ health care utilization, behavior, attitudes, knowledge, skills, and to some extent even metabolic control [16-18].

The benefits of electronic communication used by patients with diabetes, their relatives/caregivers, and health professionals were recently reviewed [19]. Although such methods show promise regarding improved diabetes care, few significant long-term effects on main outcomes could be found. Nevertheless, patients with poor metabolic control, greater use of health care services, higher motivation, and/or less experience with diabetes treatment benefited more. A few studies even demonstrated improved quality of life, although in most studies there was little focus on the patient perspective.

In light of these findings, it could be questioned why there are only a few Web 2.0 systems in routine clinical use in diabetes care [2,19-25]. At least three reasons can be identified. The first is that the process of system introduction requires active contributions from clinical professionals with experience from the present care process in order for them to play an optimal role in the improvement of care. However, most health care professionals have had little computer training in either their basic education or their professional life [26]. A second explanation is that, while young patients may already be sharing personal health information online, few health professionals are presently familiar with the rapidly emerging social networking tools on the Internet. A third reason is that when patients access information by themselves, some practitioners may experience this as a source of irritation [27]. Accordingly, a need for close collaboration between health care professionals and system developers has been increasingly pointed out [25]. In particular, the significance of practitioners’ attitudes towards computer use in nursing and in patient education has been emphasized [28,29]. Indeed, the integration of Web 2.0 resources into routine care may require iterative inclusion of the perspectives of both health professionals and patients [21]. This paper reports views and voices from a sample of experienced care providers in two Swedish units for pediatric diabetes care.

The specific aim of the study is to explore health care practitioners’ attitudes towards the introduction of a local Web 2.0 system tailored to young type 1 diabetes patients and their parents, and to seek opportunities and barriers related to introduction of such systems into clinical practice.

## Methods

### Process of Care

In Sweden, all children and adolescents with diabetes are treated by hospital-based pediatric diabetes teams consisting of nurses, nurse specialists, physicians, dieticians, social welfare officers, and/or clinical psychologists [30]. Clinicians meet their young patients and their parents at the onset of the disease when the patients are hospitalized, and continue to work with them as outpatients for many years. The process of care and the treatment policy have been described elsewhere [7,13,30]. Participants in the present study comprised two such diabetes teams at pediatric clinics situated in south-eastern Sweden which treat geographic populations of approximately 200 and 250 patients, respectively, below the age of 19 years.

### Web 2.0 Portal

**Figure 1:**
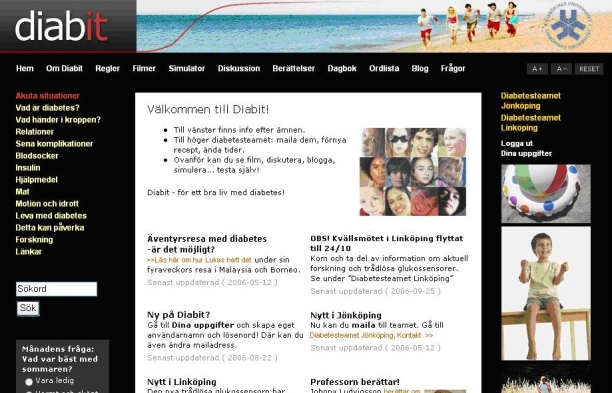
Screenshot of the portal displaying links for specific diabetes information (left); local diabetes team services, news, and personalized information (right); pedagogic devices and social networking functions (top)

During the spring of 2006, the research group and the two participating diabetes teams launched an Internet portal with specific diabetes-related information and social networking functions for patients and parents (Figure 1). Social networking functions provided laypersons with the possibility of being guided to information by apomediaries (other users on the site), which meant that the role of staff members who acted as intermediaries between users and information became less involved [6]. Thus, the content was designed for use by children, parents, and clinicians who belonged to the local patient community of the two hospitals. It included some 200 Web pages of text, education videos, and online simulation software described elsewhere [21,31]. The portal also provided general information about the diabetes teams and their services, as well as a messaging service for medical prescription renewal, appointments, and open questions.

Before launch, the portal gradually developed from a design model to the Web 2.0 prototype piloted in 2005 [32]. Thus, the user-centered design process for the portal and its contents included iterative sessions conducted over a long period of time with patients and parents, as well as the diabetes teams involved [21,32]. Specific diabetes-related information on 13 main topics, divided into 99 subtopics/Web pages, was written by an author group consisting of a nurse, a physician, and a dietician. Each section was revised by other multi-professional groups, signed by the professionals involved, and edited in a Web publishing system. In addition, each group of professionals summarized important basic information in plain speech and included their photo presentations, contact information, etc. Thus, all members of both diabetes teams participated in developing the content.

### Study Population and Methods

Through participation in previous user meetings, elaboration work, and individual test use, the interviewees had been informed about the design and functions of the portal. The present study was conducted as a baseline measure before the clinicians started using the portal in their routine practice. The interviewees (with one exception) had not met any patients or parents whom they knew had used the portal.

Considering the explorative aim of the study, we used an inductive approach to construct an interview guide with questions we believed would provide information concerning the research issues. The guide asked questions about general attitudes towards using information technology in health care, related computer skills and use of computer aids at work, perceived possibilities and motivation to participate in the elaboration of the portal, and expected consequences for clinicians and patients, both pro and con.

Of the 23 active members of the two diabetes teams, 20 were interviewed, including seven doctors, eight nurse specialists in diabetes, one nurse, two dieticians, and two social welfare officers. Two members of the research group and one person who did not agree to the interview were excluded. The interviewees had been working at the clinic for eight years on average (ranging from 1-24 years), and the majority were female. After participants gave their informed verbal consent, audio-recorded qualitative telephone interviews were conducted in August and September 2006. The interviews were semi-structured. The interviewees could raise issues themselves, and they were given time to develop answers in response to their interviewer. Follow-up questions were asked in an attempt to receive more in-depth answers. On average the interviews lasted for 30 minutes.

### Analysis

We analyzed the interviewees’ experiences within the context of culture using a recent form of phenomenology developed in American nursing studies [33]. The interviewer, a sociologist with experience in interviewing health care staff but none working in health care, analyzed the data. She did not start studying the use of information technology (IT) in patient care until the analysis had been completed. The other members of the author group were two physicians/researchers and one nurse/PhD student. This group had extensive experience in clinical diabetes care, clinical research, and medical informatics. They commented on the results in discussions that took place during the analysis process. As one researcher conducted the analysis, quotations from the interviewees’ statements were used throughout the process to facilitate validation of the findings by the other members of the research group.

In order to structure the data, the tapes were transcribed verbatim. The interviewer read the transcriptions while listening to the audio-recorded interviews and made a few corrections. Throughout the analysis, each of the staff categories, namely doctors, nurses, dieticians, and social welfare workers, was considered separately. First, the interviewer broadly categorized the issues that were discussed, which to a large extent comprised the research questions. Next, she coded all text in the categories line by line according to substantive content [34], and the codes were kept within their context. The codes were collected into themes which had emerged from the interviews, and these themes constitute the different sections in the results. Writing started early, using the first categorization for a horizontal analysis of the different themes in all interviews from the same staff category [35]. The line-by-line coding was intended as support for interpreting the meaning of the different comments.

## Results

All interviewees reported previous computer and Internet use at home and at work. In most cases, the attitude toward extended use of computers was positive. Problems were attributed to becoming familiar with the portal, implying that interviewees thought they needed to learn more about the workings of the portal. No major differences in attitudes towards using computers, the Internet, or a Web 2.0 portal were found between the different staff categories or clinics, although some differences were observed concerning obstacles to, and opportunities for, using the portal as a means of support in their work. All interviewees participated in the collection of information materials for the portal, including the development of texts and the review of texts written by others. Limited time, lack of skills in Web design, and insufficient information about the writing process were reasons why some interviewees expressed dissatisfaction with their contributions. The respondents participated in accordance with their skills, and no one reported that the work overwhelmed them. Most interviewees were satisfied with the way the portal turned out, and one interviewee said, “I don’t think we’ve ever done things this way and I think it was really nice that so many could be involved in it”.

Thus, despite different experiences with the writing process, a sense of community was reported after working with the site. Moreover, the clinicians were confident that the portal’s use in diabetes care would extend beyond the clinics, in addition to being a part of the internal routine of the clinics. Interestingly, most interviewees reported being prepared to keep working on the development of the portal and expected to maintain an active role, as expressed by the following participant:

Well, if it’s something we’re going to work with in the future, then of course I want to be involved and participate in it, of course, but ... in some way or another ... so that it seems practical to me too.

### Expectations of Web 2.0 Portal Use in Diabetes Families

Expectations varied regarding the impact a Web 2.0 portal would have on the everyday lives of patients and their families. Several interviewees offered optimistic comments:

I think it will probably be of great importance to patients to be able to gain access to information so easily .... And anyway, most children and adolescents are familiar with the Internet today ....

Others were less hopeful concerning the use of Web 2.0 services. One reason for this was that parents and adolescents were presumed to have different needs, and it might therefore be difficult to design the portal so that it would appeal to all users. Another perceived risk was that only those who were already well informed would use the portal. In accordance with the low expectations of some of the interviewees, others felt that those who were not very interested in Web services in the first place would not become more interested just because of the introduction of new media. One interviewee said, “Many of our patients aren’t very interested in reading at all ... and then when this reluctance is combined with something new, well I don’t know, it’s a problem”.

Speculating on the prospects for success of the Web 2.0 portal, clinicians were of the opinion that simply providing information on a website might not be enough to enable all patients or their families to integrate the information available there and increase their self-efficacy. One interviewee explained it this way:

And I think there are so many different factors that make it possible for a person to take in information, and I mean ... how the person feels and what things are like in the family, and how easy it is for the person to understand and, well, there’s a lot contributing to what support the person has from those around him.

Accordingly, peer-to-peer communication online was specifically noted as being a key function of the portal, since contact between peer families could facilitate living with diabetes, as was suggested by one interviewee who commented that, “Maybe they ... will receive good suggestions from other patients, if they have an opportunity to discuss it”.

A few interviewees emphasized the importance of maintaining some professional control over the site in order to reduce the risk of communicating harmful advice or passing along incorrect references concerning the management of diabetes. One interviewee expressed concern over the risk of young people revealing too much personal information about themselves and then regretting it later.

Despite the proposed benefits, most interviewees did not expect that the portal would initially be used very much for peer-to-peer contact. Some interviewees thought that social networking functions would probably be most appreciated by the parents of young children with problems, since they were expected to require more support. Others thought that adolescents would be the most frequent users, since they are the group most familiar with the media. One interviewee said that, “A young person may have a lot of questions he might not want to talk about with either his parents or the diabetes nurse, but he may be willing to talk with a friend who’s in the same situation”.

However, some interviewees thought that teenagers would ask for the ability to make peer-to-peer contact and then decide against doing so:

But even when you arrange something, they don’t always come anyway .... You have to catch adolescents on the run in some way.

A common idea which emerged from the interviews was that access to a properly updated portal might encourage some patients to take an active role in learning more about their disease by searching for news and extending their search to other websites. One person said, “It can be a way to get information about things a person doesn’t get around to asking the doctor about, and I think that can be good”.

Another view expressed in the interviews was that, during face-to-face interaction and telephone contact with team members, patients received more complete information, since they could ask questions and receive their answers directly. Team members also provided information the patient did *not* ask for, and they took different circumstances into consideration. “You can hear how they feel from their voices and the like,” one interviewee said. Thus, direct contact helped staff to provide personalized advice that was adapted to the receiver’s needs at a particular point in time. A possible outcome of this is expressed in the following: “My idea would be maybe to add more information-based questions on this site [the portal] and answer questions about treatment over the telephone”.

Different complementary ways of providing information are described below:

They can read and take in information, and they can get it when they want it and at the pace they want, and if they wonder about something more, they can supplement that information by calling or asking questions at their next visit here. I think that’s good.

Most interviewees presumed that all families had a computer and Internet access, and that it was natural for families to get and provide information about diabetes online. However, one interviewee stressed the following:

This can’t be the only method available ... so that if you need to get information, you have to do it yourself, and you have to do it on the Internet, period.

If everyone does not have access to the information, the portal is not a common source of information, and if the portal should become the primary source of information, this might have negative consequences for those without Internet access.

Other reasons for caution that were mentioned by interviewees were the risk that patients would find information that frightened rather than motivated them; that they would develop false hopes about their chances of getting rid of their disease; or that some parents might “escape” into technical information on the Internet when they could not bear the fact that their child had a serious disease. Another risk identified was that patients could believe they were so well informed by the portal that they would not keep appointments with the diabetes team, or they would try their own treatment and fail.

### Use in Clinical Diabetes Team Practice

According to most of the interviewees, one important function of the Web 2.0 portal was that it facilitated closer interaction between diabetes teams and families. In particular, it was expected that patients having long-term experience with diabetes would be more comfortable asking certain questions via the Internet and that the portal could even stimulate families to contact team members. According to the following interviewees:

If you feel uncertain and don’t even want to call and make contact to find out what a staff member can do to help, then you can log on to this site so that you can get information you may need. At the very least, you can make contact.

It’s not only the case that there’s a child that has diabetes. There’s a mother and a father who have jobs and take part in leisure-time activities, and maybe there are siblings. They have a very full schedule. They might not be able to reach us during the day when we’re here, but when they come home in the evening and things have calmed down, maybe they can send a message or a question, or maybe say that they need some [diabetes] device.

However, several interviewees also pointed out that it was unclear whether current legislation permitted email contact with patients, while others were uncertain about this but expected email communication to be safe. One interviewee stated, “I can do my banking online, so I certainly should be allowed to communicate with patients.

Other expected benefits of the portal were more traditional Web 1.0 functions (ie, providing information). Newly updated diabetes information would be available to families at any time (eg, when something unexpected happened or whenever information was needed during the regular three-month period between visits). Options for repeating information received in person at the clinic, as well as for updating old information, were also mentioned. For instance, children with early onset diabetes need to learn about their disease while growing up in order to become independent, since when they are young, their parents have more knowledge about the disease. The interviewees also emphasized, however, that the purpose of Web information was not to have families take on the responsibility of obtaining all information by themselves.

Since the information on the portal available from each respective diabetes team was identical to information provided at the clinic, it was described as “familiar”. Several interviewees expressed that a locally shared source of reliable information, such as references to verified websites, would be a great support to their work with patients, assuming that it was regularly updated. It could also be used by new team members or other staff, as commented on in the following: “I think this is a function the portal could provide to make it easier for those who don’t work much with diabetes. That function would be to provide advice that doesn’t deviate too much from what they receive from the diabetes team”.

Conformity of information could create a sense of security for families and also for relatives, friends, and school staff who want information. It was also thought that better informed patients would interact more often by asking more questions that would stimulate clinicians to keep up to date with news about diabetes care. Supplying patients with information about the responsibilities of the clinic was perceived as a challenge to the diabetes team: “For us it can also be a way to be a little more on the ball because it’s out there in public view”.

Another expected benefit of the Web 2.0 portal was its use in support of routine clinical checkups. The portal was described as a means of achieving a more informative and effective clinical encounter, which touches on the topic of time. Lack of time and how to deal with this problem was an issue often raised during the interviews. Several interviewees expected the portal to save time in the execution of some routine tasks and when providing general information. A few interviewees related the following:

I thought it sounded good because it could supplement what we don’t have time for during visits to the clinic .... instead of having to call ....

Most interviewees thought that extended use of email would save time and increase flexibility. Since patients need to be able to talk with a health care worker in acute situations, email was not perceived to be the best option in every situation. In addition, one interviewee stated that he did not want to be unexpectedly overwhelmed by email:

I want to know when my contact with patients will take place as I would if, for example, I had fixed email hours. Currently, we have fixed telephone times.

## Discussion

### Main Findings

We found that pediatric practitioners reported a range of positive attitudes towards the introduction of a local Web 2.0 portal for young diabetes patients in their clinical practices. This is in contrast to attitudes of “resistant compliance” to computers in routine work reported in some other settings [28,36]. The findings are particularly interesting in light of the fact that the practitioners reported having been unfamiliar with Web 2.0 technology before the study and that all the legal aspects of Web 2.0 use at the clinics had not been settled (eg, the legal aspect of email communication with patients).

As diabetes treatment largely consists of daily self-care, enhanced patient education and support remain essential to pediatric practitioners’ efforts to improve quality of care [8,13,30,31]. Thus, one explanation for their positive outlook is probably the interviewees’ early participation in a collaborative design process. Their multi-professional development of information also seemed to ensure that a unified message about the treatment policy was provided by all members of the care team. Although some practitioners felt constrained by limited time and a lack of skills in Web design and publishing, participation may have produced a reciprocal learning process and a sense of community with respect to the portal. It may be that this approach led to a willingness to integrate the portal into routine care, as well as to engage in further developmental work. Our findings highlight the need for general efforts to enhance the education of clinical practitioners and others involved in the management of childhood chronic diseases regarding the use of Web publishing, social networking, and other Web 2.0 resources [26].

Constructive attitudes could also be attributed to the fact that a local Web 2.0 portal was perceived as potentially beneficial for both patients and staff. Other studies have indicated that two significant outcomes of using a Web 2.0 portal in routine care are the empowerment of patients and facilitation of work due to time-saving, simplified routines [37,38]. Confidentiality and patient integrity were also generally perceived to be managed satisfactorily by the system. Some practitioners suggested that patient trust could be enhanced by making certain that information supplied by the portal matched the information provided during personal visits to the clinic. It was furthermore inferred that the presence of local diabetes team members increased a patient’s sense of security and stimulated greater interest in the portal. Quality and trust issues regarding online health resources have been the focus of much discussion [39]. In a chronic disease setting, it could be that a balance between information supplied during a personal meeting and information acquired via a local Web 2.0 portal may result in the optimization of efficiency, quality, and trust.

Practitioners expressed an open attitude and positive expectations towards the idea of more informed patients and parents, as well as the support of apomediation in online peer networks. However, they also expressed doubt concerning the progress and actual use of this section of the portal. Internet support groups have, however, reportedly improved parents’ relationships with their children with special needs [40]. In addition, results from studies of diabetes-related Internet support groups seem encouraging, but few population studies have been conducted [41]. As of today, few real-world diabetes teams offer their patients online networking systems.

With regards to the issue of control, practitioners seemed to accept the loss of direct control over information when patients began to inform themselves by using apomediators online. Modern diabetes care involves teamwork which aims at developing empowered and well-informed patients. Participation in, openness to, and problem-based learning about the discipline of self-care have been regarded for many years as essential elements of pediatric diabetes care [8,30,31]. If social networking functions are actively used by families, one consequence might be their increased control over online information, since it is derived from peers in the community who have experienced similar treatment. Moreover, patients using the portal could be expected to benefit from increased knowledge about when to contact their diabetes team, when to seek information, and what to seek at any particular point in time.

Because it is difficult to design a website that will attract patients and parents with different proficiency and preferences, some interviewees feared that the site might be used primarily by those who were already well informed. This perceived risk seemed to stimulate the clinicians desire to “keep the site alive”, and they expected that this would result in new work tasks (eg, to add news and updates from the local practitioners). Importantly, some pointed out that the portal cannot replace personal contact. They emphasized that individualized telephone contact or face-to-face interaction, particularly in emotionally difficult situations and when complex issues are involved, will remain necessary. Finally, another source of anticipated loss of control was that, with the clinicians’ work routines available online, patients could more efficiently question the clinicians’ planning of services.

This study has some important limitations that need to be taken into account when interpreting the results. The study does not provide information about the attitudes of care teams, other than those involved during the design process. It is not possible, based on the data, to predict if the specific functions of the Web 2.0 portal will produce benefits during routine use, even though the practitioners in this study thought that the disadvantages, if any, would be outweighed by the advantages. In addition, because the study was performed using qualitative methods for data collection and analysis, it is not possible to quantify the attitudes observed. For instance, both generally positive attitudes and attitudes which expressed some doubt regarding the support of apomediation were recorded, but this study cannot quantify the proportions of these views. A strict, independent categorization of data by a second researcher might have further strengthened the validity of the results.

For future research, larger studies are warranted which would take into account the views of practitioners, as well as diabetes patients and their families, on the routine use of Web 2.0 portals, and such studies should include the collection of both qualitative and quantitative data. Investigation of attitudes towards implementing similar systems in the care of adults with chronic diseases are needed as well. Little is known regarding predictors for success (eg, comparisons with respect to benefits and pitfalls between patient-driven, peer-to-peer online networks and organization-driven networks monitored by professional “experts” have yet to be carried out). As every patient community has its own characteristics and needs, there is probably no such thing as a “one size fits all” model. Finally, the extent to which increasingly well-informed patients might stimulate creative dialogues remains to be explored, whether these take place between patients and care teams or within care teams themselves, with the aim of attaining coherent views and increased quality of care [13].

### Conclusion

We found a range of positive attitudes towards the introduction of a local Web 2.0 portal and perceived benefits for patients of experienced clinical practitioners working with young diabetes patients. These findings contrast with previous reports and may hypothetically be associated with the early and active involvement of clinicians and their patients in the development work.

The implications of the results for future implementation of Web 2.0 systems in health care include the need for education of clinical practitioners in the use of Web 2.0 and the understanding that collaboration with all user groups is beneficial for developing the site. The findings are encouraging for the development and implementation of Web 2.0 resources as part of the care of young patients with chronic diseases, in particular those suffering from type 1 diabetes. There might also be important implications for the care of adult patients with diabetes and for other diagnosis groups as well.

## Abbreviations

IT: information technology
